# Projecting seismicity induced by complex alterations of underground stresses with applications to geothermal systems

**DOI:** 10.1038/s41598-021-02857-0

**Published:** 2021-12-07

**Authors:** M. Cacace, H. Hofmann, S. A. Shapiro

**Affiliations:** 1 Helmholtz Centre Potsdam GFZ-German Research Center for Geosciences, Potsdam, Germany; 2 Earth Science Department, Freie Universität Berlin, Berlin, Germany

**Keywords:** Seismology, Natural hazards

## Abstract

Seismicity associated with subsurface operations is a major societal concern. It is therefore critical to improve predictions of the induced seismic hazard. Current statistical approaches account for the physics of pore pressure increase only. Here, we present a novel mathematical model that generalises adopted statistics for use in arbitrary injection/production protocols and applies to arbitrary physical processes. In our model, seismicity is driven by a normalised integral over the spatial reservoir volume of induced variations in frictional Coulomb stress, which—combined with the seismogenic index—provides a dimensionless proxy of the induced seismic hazard. Our model incorporates the classical pressure diffusion based and poroelastic seismogenic index models as special cases. Applying our approach to modeling geothermal systems, we find that seismicity rates are sensitive to imposed fluid-pressure rates, temperature variations, and tectonic conditions. We further demonstrate that a controlled injection protocol can decrease the induced seismic risk and that thermo-poroelastic stress transfer results in a larger spatial seismic footprint and in higher-magnitude events than does direct pore pressure impact for the same amount of injected volume and hydraulic energy. Our results, validated against field observations, showcase the relevance of the novel approach to forecast seismic hazards induced by subsurface activities.

## Introduction

An earthquake—be it tectonic or man-made—is a sudden release of elastic energy upon an excess of shear stress relative to the shear strength of a fault^1^. In the context of induced seismicity (i.e. seismicity associated with subsurface operations), little debate exists on the main co-factors that lead to fault instability, which include the tectonic in-situ stress, local geology, pore pressure- and temperature changes, and stress redistribution from event–event interactions and (a)seismic slip^2^.

Traditionally, pressure variations have been considered the main driver of induced earthquakes, which has inspired extensive literature on the induced seismic hazard with the aim of correlating expected seismicity to pore pressure variations within a probabilistic seismic-hazard framework^3,4,5^. The advantage of stochastic analysis is that once calibrated to available catalogues, it can provide near-real-time forecasts of seismic hazards that can be fed into advanced traffic light systems (ATLS)^6,7^. This information can be helpful in pinpointing correlations between induced seismicity and operational parameters, which can be controlled and optimised in the field^8^.

Common practice in reservoir studies is to correlate induced earthquake magnitudes to the total injected fluid volume^9^. The assumption is that the rupture size of an induced event correlates with the stimulated rock volume and that this volume can be controlled during injection^10^. This concept has been critically revised in recent works, which argue that tectonics and local geology play concomitant roles in induced earthquakes^10,11^.

A growing volume of information from cases of induced seismicity highlights the importance of mechanisms other than pore pressure variations. Seismicity ahead of the pore pressure front was monitored in meso-scale injection experiments^12,13^ and was interpreted as the result of induced poroelastic stress and aseismic stress transfer^14,15^. Thermal stresses associated with cold-water re-injection have also been found to result in an increased seismic risk in geothermal systems^16^. Recent experimental findings have revealed that injection-induced seismicity depends both on magnitudes of fluid-pressure build-up and on imposed pressurization rates^17,18^.

These observations require a critical revision of our understanding of the controlling mechanisms of induced earthquakes. An increasing number of field studies have challenged the validity of log-linear magnitude scaling correlations between expected induced earthquake magnitudes and cumulative injected volume, with the most-discussed example being the 5.5 Mw Pohang earthquake^19^, whose magnitude exceeded estimates based on the total injected volume by a factor of over 800^20^. At the Geyser geothermal field, induced seismicity has been linked to thermal contraction from steam withdrawal and associated thermoelastic stresses^21^. The stimulation performed at the Groß Schönebeck site offers another example in which a large volume of water injected at high flow rates triggered only micro-seismicity^22^.

In the present work, we outline a novel mathematical model that integrates the details of reservoir physics into a statistical framework for induced seismicity. Our study generalises and departs from existing approaches in its application to arbitrarily complex injection/production protocols, and it accounts for the non-linear thermal, hydraulic, and mechanical response of the stimulated reservoir. Our model represents a substantial step towards integrating reservoir physics into a probabilistic hazard-assessment framework with the goal of advancing the forecasting of induced and triggered earthquakes.

## Modified Gutenberg–Richter (GR) statistics for induced earthquakes

The classical GR statistics for induced seismicity is described by the following equation^23,24^:

1 $${\mathrm{log}}_{10}[{\mathrm{N}}_{\ge \mathrm{M}}(\mathrm{t})]={[\Sigma }_{0} +\mathrm{ \delta \Sigma }(\mathrm{t})]-\mathrm{bM}$$

In Eq. (1), the seismogenic index (SI), ${\Sigma }_{0}$, quantifies the seismo-tectonic activity of a given geological site—that is, the potential of a particular site to produce a given level of seismic activity in response to a given perturbation of the internal stress and pore pressure state^23–26^. For instance, for fluid injection induced seismicity, ${\Sigma }_{0}$ can be computed as the logarithm of the number of induced seismic events of positive magnitudes per unit of injected volume^26^. $\mathrm{\delta \Sigma }(\mathrm{t})$ should be considered a time-sensitive correction that accounts for the effects from reservoir operations. By assuming that pore pressure relaxation triggers instability, the number of events (${\mathrm{N}}_{\ge \mathrm{M}}(\mathrm{t})$) scales with the total injection volume (${\mathrm{V}}_{\mathrm{fluid}}(\mathrm{t})$), and the classical scaling between induced event magnitude and net injected fluid volume^9,10,23,24^ can be derived as:

2 $$\mathrm{\delta \Sigma }\left(\mathrm{t}\right)={\mathrm{log}}_{10}\left[{\mathrm{V}}_{\mathrm{fluid}}\left(\mathrm{t}\right)\right].$$

In the present model, we generalise Eqs. (1) and (2) to account for arbitrary physics, which are considered relevant in induced seismicity. We consider Mohr–Coulomb failure to be a valid criterion for earthquake occurrence and use variations in frictional Coulomb stress ($\mathrm{\delta FCS}$) at critically oriented faults to estimate the number of earthquakes at a given location and time. Seismic instability is therefore determined by resolved variations with respect to an undisturbed tectonic stressing state of FCS, with positive changes favouring the generation of seismic events and negative values contributing to fault stabilisation:

3 $$\mathrm{\delta FCS}=\mathrm{\delta \tau }-\upmu (\mathrm{\delta \sigma }-\mathrm{\delta p})$$

In Eq. (3), $\mathrm{\delta \tau },\mathrm{ \delta \sigma }$, and $\mathrm{\delta p}$ are variations in shear stress, normal stress, and pore pressure, respectively, and $\upmu$ is the friction coefficient. Please note that we assumed that compressive stress is positive when deriving Eq. (3) and that cohesive force and the friction coefficient are considered constant (though these last assumptions can be easily disregarded).

The total number of induced earthquakes can thus be defined as the product of the volumetric integral of the probability that each fault will fail seismically (the probability density function of criticality) times the density concentration of faults in the reservoir (see also Supplementary Information 1 for its derivation). We express this probability as the integral of resolved $\mathrm{\delta FCS}$ over the entire reservoir domain:

4 $$\mathrm{\delta \Sigma }(\mathrm{t})={\mathrm{log}}_{10}[{\int }_{\mathrm{V}}\frac{\mathrm{SM}[\mathrm{\delta FCS}(\mathrm{x},\mathrm{t})}{\mathrm{sin}(\uppsi )}\mathrm{dV}]$$

In Eq. (4), $\mathrm{S}$ is the uniaxial storage coefficient, $\uppsi$ is the friction angle, and $\mathrm{\rm M}[\mathrm{\delta FCS}(\mathrm{x},\mathrm{t})]$ is the minimum positive monotonic majorant of $\mathrm{\delta FCS}(\mathrm{x},\mathrm{t})$^27^. Please note that in the case of a non-monotonic rock stimulation, the use of $\mathrm{\rm M}[\mathrm{\delta FCS}(\mathrm{x},\mathrm{t})]$ implies that an earthquake can be induced at a given location if resolved FCS variations exceed the maximum level attained during the evolution of the system. This enables to account for the well-established Kaiser effects in rock mechanics^28^.

From Eqs. (1) to (4) we derive the total number of induced earthquakes above a certain magnitude M and at a given elapsed time as:

5 $${\mathrm{N}}_{\ge \mathrm{M}}(\mathrm{t})={10}^{({\Sigma }_{0} -\mathrm{ bM})}{\int }_{\mathrm{V}}\frac{\mathrm{S M}[\mathrm{\delta FCS}(\mathrm{x},\mathrm{t})]}{\mathrm{ sin}(\uppsi )}\mathrm{dV}$$

By taking the derivative of Eq. (5) with respect to time, we can derive the following expression for the seismicity rate, that is, the number of events over time:

6 $${\frac{\mathrm{d}}{\mathrm{dt}}\mathrm{N}}_{\ge \mathrm{M}}(\mathrm{t})={10}^{({\Sigma }_{0} -\mathrm{ bM})}\frac{\partial }{\partial \mathrm{t}}{\int }_{\mathrm{V}}\frac{\mathrm{S M}[\mathrm{\delta FCS}(\mathrm{x},\mathrm{t})]}{\mathrm{ sin}(\uppsi )}\mathrm{dV}$$

Combining the above equations and assuming that the scaling of fault sizes obeys a GR frequency magnitude distribution yields the following formula for the expected maximum magnitude ( <${M}_{max}(t)>$) within a given statistical sample (see also Supplementary Information 1 for its detailed derivation):

7 $${<M}_{max}(t)>=\frac{1}{b}({\Sigma }_{0}+{\mathrm{log}}_{10}[{\int }_{\mathrm{V}}\frac{\mathrm{S M}[\mathrm{\delta FCS}(\mathrm{x},\mathrm{t})]}{\mathrm{sin}(\uppsi )}\mathrm{dV}])$$

Equations (4) and (7) generalise the classical SI approach to induced seismicity^23,24^ by accounting for any physical process responsible for fault destabilisation, which is here expressed in terms of variations in resulting FCS. FCS variations can be computed by numerically solving for fluid mass, internal energy balance, and thermo-poroelastic reservoir response, as is routinely done in applied reservoir studies (see Supplementary Information 2).

**Figure 1:**
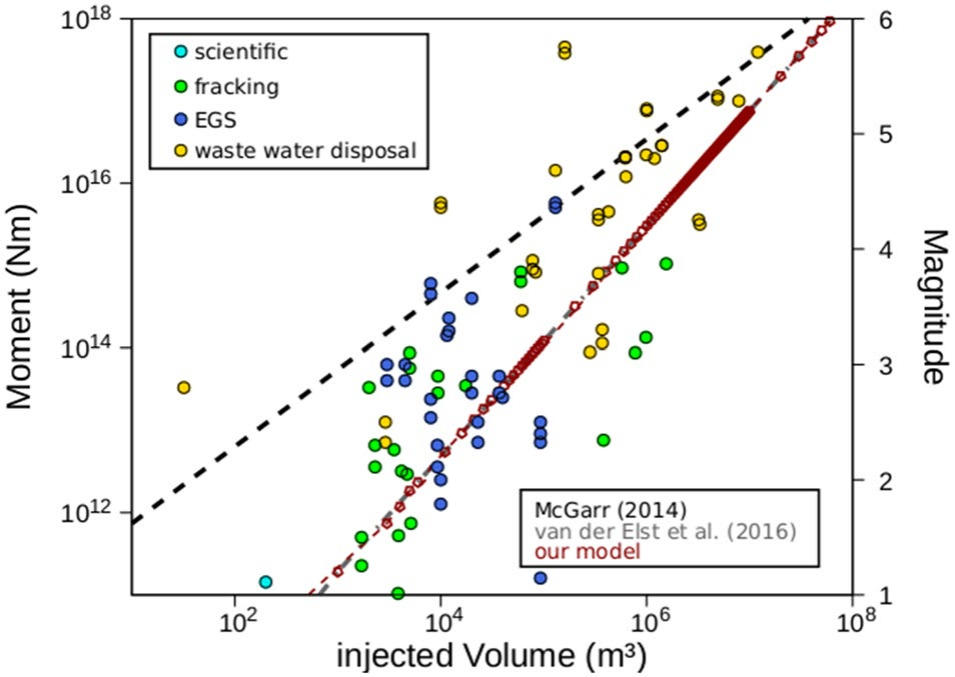
Maximum observed magnitudes and seismic moment as a function of the total injected volume for a number of site applications. Data were taken from^9,29–31^. The predictions from this study (Eq. 7 in the main text) are shown by the red squares. The black curve is estimated by^9^ (G = 30GPa) and the grey curve by^10^ using the same seismogenic index (${\Sigma }_{0}=-1.8$) and a constant b value (b = 1) as in our model.

For monotonic injection of an incompressible fluid within a linear poroelastic reservoir, we can approximate $\mathrm{\delta FCS}$ in terms of variations in pore pressure (Eq. 3). In this case, pore pressure is a monotonically increasing function of elapsed time: $\mathrm{M}[\mathrm{\delta FCS}(\mathrm{x},\mathrm{t})]=\mathrm{M}[\mathrm{p}(\mathrm{x},\mathrm{t})]=\mathrm{p}(\mathrm{x},\mathrm{t}).$ Therefore, Eq. (7) reduces to the classical SI formalism and to a log-linear scaling between maximum expected magnitudes and net injected fluid volume^10^ (Fig. 1). The classical log-linear correlation between maximum expected induced magnitude and net injected volume naturally arises from the new model as a limiting case for scenarios in which the contribution from thermo-poroelastic stress transfer can be deemed as negligible.

## Induced seismicity in geothermal systems

### Effects of injection rates and protocols

Observations derived from field applications and laboratory studies indicate a correlation between induced-seismicity rates and fluid-pressurisation rates^17,18^. In this paragraph we show that rates of $\mathrm{\delta FCS}$ map directly onto seismicity rates (Eq. 6), thereby enabling to control for the probability of the occurrence of a seismic event at a given time. More information about the model set up, boundary and initial conditions are provided in Supplementary Information 2.

**Figure 2:**
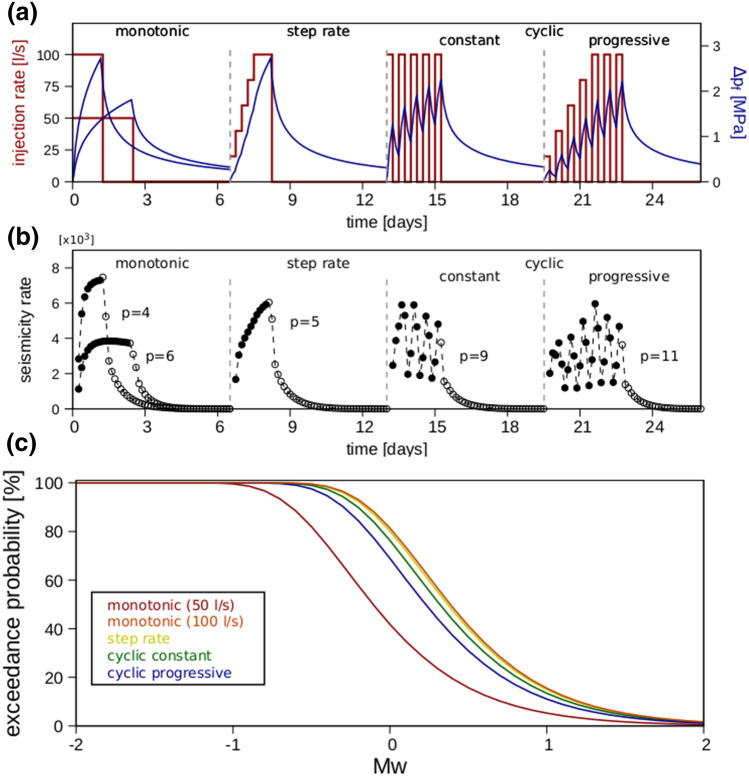
Effects of injection protocols on induced seismicity rates and magnitudes. (**a**) Injection rates (red curves) and computed wellhead overpressure (blue curves) for the tested injection protocols. (**b**) Resulting seismicity rates and the power-law exponents (Eq. 8) that best fit the post-injection decay curves (empty circles). (**c**) Cumulative exceedance probability of a given magnitude Mw (Eq. 9). Each scenario lasted 6.5 days and used the same amount of net injected fluid volume (10,800 m^3^) and hydraulic energy (Figure S1). Different cases (upper and middle panels) have been shifted in time to ease visual comparison.

Figure 2 reveals how injection protocols can affect the induced seismicity, both in terms of rates and magnitudes. In line with existing observations, we find that seismicity rate is proportional to injected pore pressure and—most importantly—to imposed pressurisation rates. In our formulation, the dynamic triggering of seismic events in space and time is dictated by the ability of the reservoir to relax induced variations in FCS, and these variations also control post-injection seismicity rates. Accordingly, modelled decay in seismicity rates at shut-in can be fitted by a modified Omori’s law^32^ in the form of (see also Supplementary Information 4):

8 $${N}_{\left(\ge M\right)}\left(t>{t}_{0}\right)= \frac{1-({\frac{t}{{t}_{0}})}^{\left(1-p\right)}}{p-1}{N}_{\left(\ge M\right)}\left(t={t}_{0}\right),$$

where ${N}_{(\ge M)}(t>{t}_{0})$ is the number of cumulative events after shut-in, ${N}_{(\ge M)}(t={t}_{0})$ describes the number of cumulative events at shut-in and p denotes the exponent of the power law distribution.

When considering monotonic injection, higher rates increase the overall seismic potential and lead to a steeper seismicity-rate curve during stimulation—followed by a more-gradual decay at shut-in (smaller power-law exponent, p)—and therefore to a higher post-injection seismic risk.

The magnitude distribution also depends on imposed injection rates, with higher rates being more likely to produce higher-magnitude events^10^. This is shown in the lower panel of Fig. 2 where we plot the obtained induced earthquake probability distributions for each protocol. The latter probabilities are computed by assuming that the occurrence of a given-magnitude earthquake can be described by a Poisson process. Therefore, the probability of exceeding a given magnitude (Mw) is given by:

9 $$\mathrm{w}\left({\mathrm{M}}_{\mathrm{w}}\right)= 1-\mathrm{ w}\left(0,{\mathrm{M}}_{\mathrm{w}},{\mathrm{N}}_{\ge \mathrm{M}}\right)= 1 -\mathrm{ exp}\left(-{\mathrm{N}}_{>\mathrm{M}}\right),$$

where $\mathrm{w}(0,{\mathrm{M}}_{\mathrm{w}},{\mathrm{N}}_{\ge \mathrm{M}})$ is the probability of having no earthquakes of a magnitude greater than or equal to ${\mathrm{M}}_{\mathrm{w}}$ within the specified time given the expectation of having ${\mathrm{N}}_{\ge \mathrm{M}}$ earthquakes whose magnitudes are greater than or equal to ${\mathrm{M}}_{\mathrm{w}}$.

Our results demonstrate that it is possible to reduce the seismic risk during both active stimulation and shut-in by controlling rates and pressure magnitudes via stepwise-controlled injection or a cyclic-stimulation strategy. The role of cyclic-injection strategies or step-rate tests in influencing seismic-hazard potential depends on (1) the duration of each cycle, (2) imposed injection- and pressurisation rates, and (3) the time needed for the induced overpressure to equilibrate between each stage (Fig. 2).

With respect to the post-injection phase, cyclic rock stimulation is characterised by significantly faster relaxation upon shut-in and therefore by higher decay rates of induced seismicity (i.e. higher p values) than is monotonic or step-rate fluid injection. Thus, given equivalent tectonic conditions (the same ${\Sigma }_{0}$) and the same injected volume and hydraulic energy (Figure S1), the seismic hazard of a cyclic stimulation is significantly smaller (especially during the post-injection phase) than the hazard of monotonic injection strategies.

Deriving generic conclusions on the influence of the discussed stimulation protocols (which should be evaluated for individual cases) lies beyond the scope of this study. However, our results—which have been corroborated by novel laboratory^17,18^ and field evidence^33^—demonstrate the importance of systematically controlling the injection protocol to minimise the seismic hazard induced by subsurface stimulation.

### Role of thermo-poroelastic stress transfer during long-term circulation of geothermal fluids

The dynamics of fluid-induced seismicity have been traditionally limited to the direct effects of pore pressure increase within the stimulated rock volume, and the effects of thermo-poroelastic stress transfer have only recently grown in importance^14,34–38^. These additional mechanisms are particularly relevant for geothermal applications over relatively long time scales of concurrent cold-water injection and hot-water production.

In our approach we can handle both long-range (quasi-)static thermo-poroelastic and direct pore-pressure-effect interactions over time and we can effectively translate these interactions into seismicity-rate changes and into the probability of the occurrence of a seismic event of a given magnitude at a given time. To further understand how thermo-poroelastic stress transfer can affect seismic hazard, we simulated a long-term circulation scenario (via simultaneous injection and production) and analysed the seismogenic stability of the system. We targeted a geothermal doublet lifetime of 30 years and continuously re-injected geothermal fluids (at 70 °C at 50 l/s) into the reservoir (at 150 °C).

**Figure 3:**
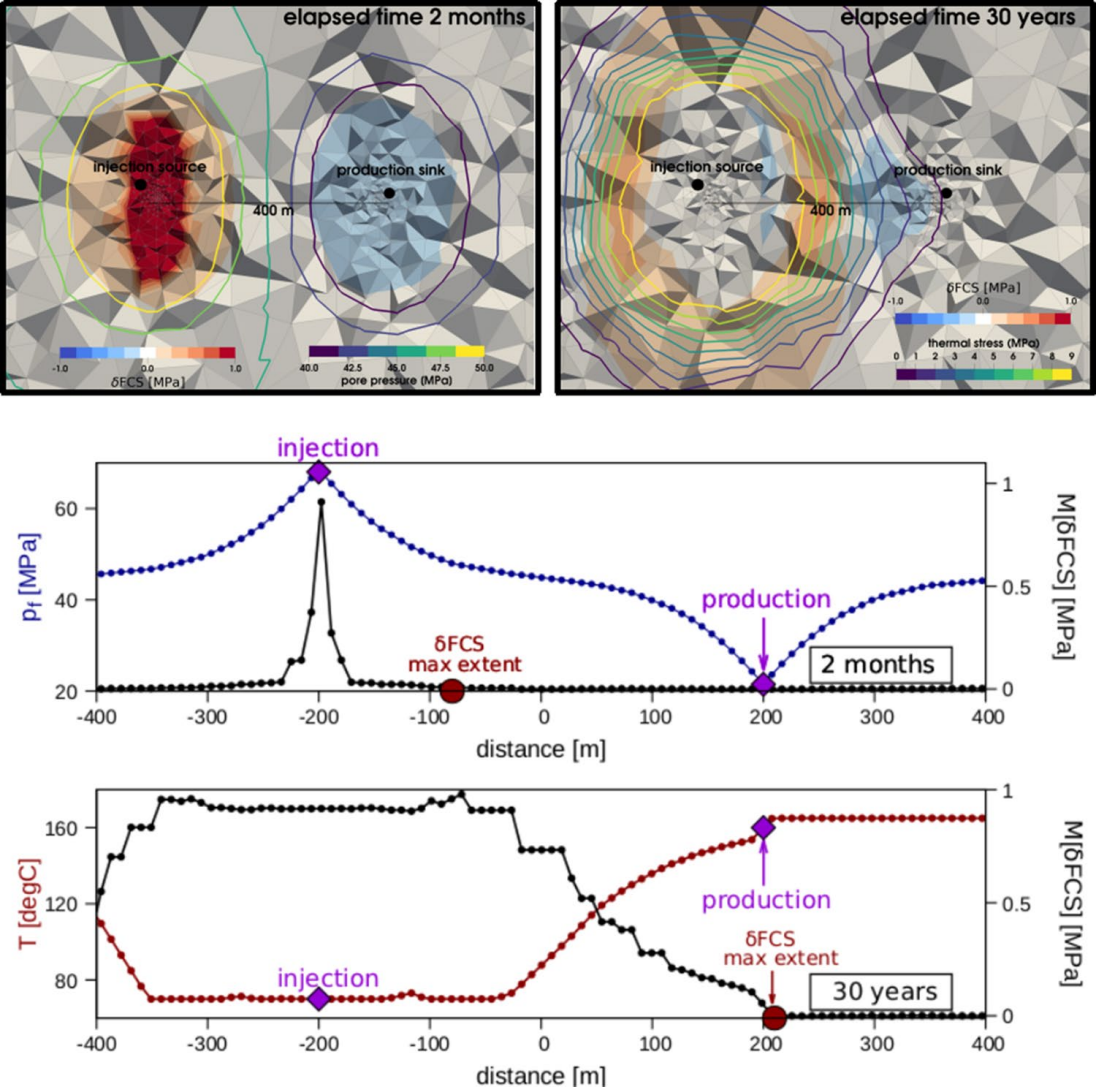
Thermo-poroelastic stress transfer and induced variations in FCS during long-term geothermal operations. Upper panels: computed variations in FCS for a thermo-poroelastic simulation of a 30-year continuous injection and production. Left: model results at the time when pore pressure equilibrated in the system (maximum extent of the pore pressure front). Right: model results at the final stage of the operations. Background colours indicate computed values of $\mathrm{\delta FCS}$, and isocountours of computed pore pressures (left panel) and thermal stresses (right panel). Lower panels: variations in computed $\mathrm{\rm M}[\mathrm{\delta FCS}]$ extracted from the 3D model along a line passing through the injection- source and production-sink. During the early stages of the system evolution, variations in FCS are bounded by the pore pressure front and show an abrupt decay away from the injection boreole that is driven by pore pressure diffusion only. Once pressure equilibrium is reached in the reservoir, variations in FCS are driven by changes in thermo-poroelastic stresses, thereby resulting in a larger seismic footprint and a more-gradual spatial decay away from the injection source.

**Figure 4:**
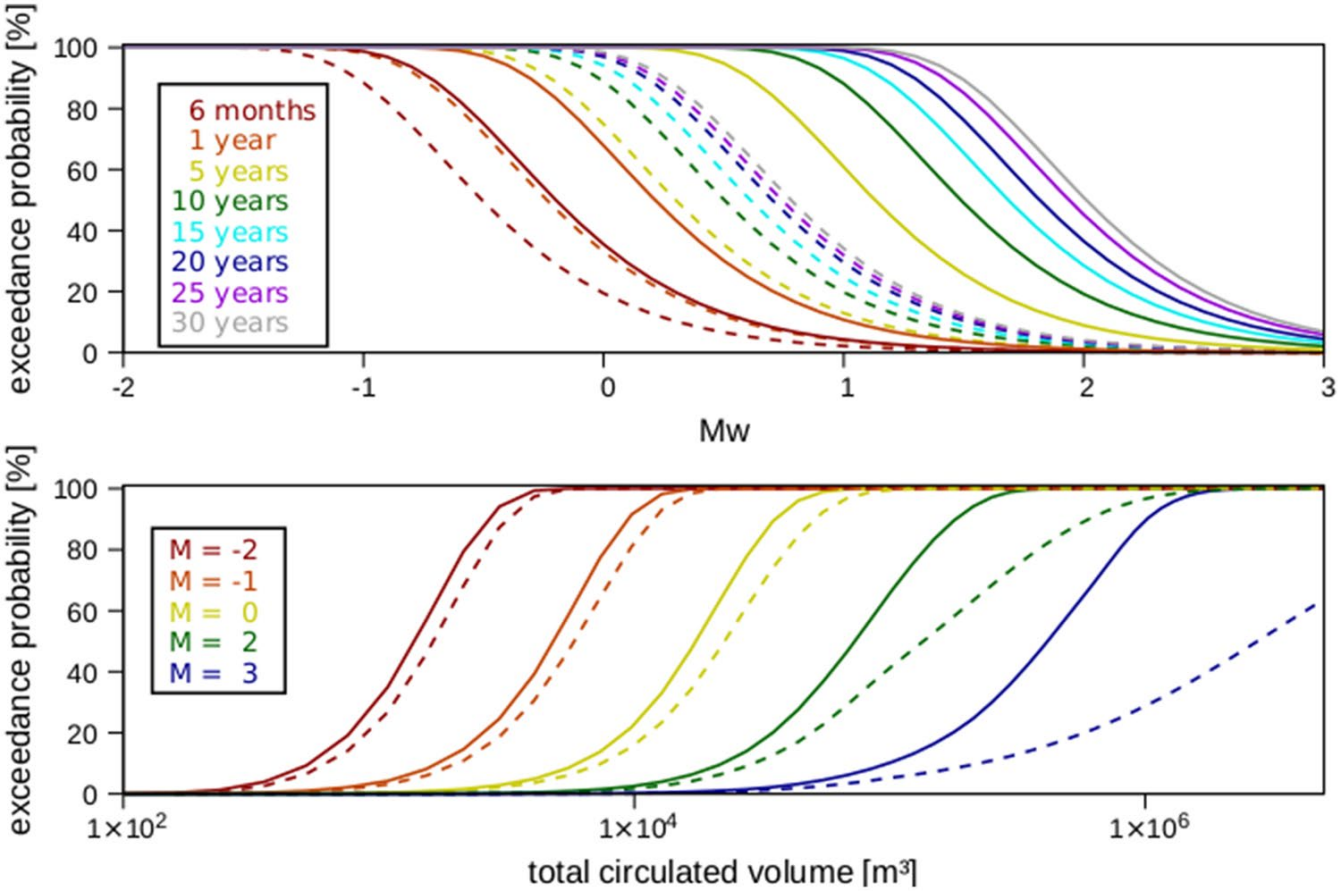
Cumulative exceedance probability of a given magnitude Mw (upper panel) and its evolution as a function of the total circulated volume (lower panel) in a geothermal well doublet during 30 years of continuous injection and production. Continuous curves indicate cases for which thermo-poroelastic effects are considered, and dashed curves indicate cases for which these additional effects are disregarded. The difference between the curves increases with time and peaks after pore pressure equilibrates in the system.

We identified two main stages in the evolution of the system: (1) a short-term regime (before pore pressure equilibrates), during which instability is driven by the induced pore pressure increase near the injection well and variations in FCS are limited by the fluid-pressurised front, and (2) a long-term regime (after pore pressure equilibration), which shows a dominance of thermo-poroelastic effects (Figs. 3, 4). During this stage, thermo-poroelastic stress variations control the geomechanical response of the reservoir and result in a larger spatial footprint of positive variations in FCS that extend beyond the pressurised front (Fig. 3).

Variations in the stress state due to coupled thermo-poroelastic stress also affect the seismicity distribution (Fig. 4). By disregarding thermo-poroelastic effects, the forecasted annual probability of exceedance (Eq. 9) is biased towards lower magnitudes for all times and/or circulated fluid volumes. The difference between the two models systematically increases with the circulated volume, thereby suggesting that cold-fluid re-injection can induce larger events than pore pressure diffusion alone, especially at later times in the system evolution. Computed differences concern mostly the highest magnitudes in the explored range (Mw > 2), thereby illustrating the significant risk of the long-term circulation of geothermal fluid, which must be accounted for in any seismic-risk-assessment- and mitigation strategy.

### Modelling of the 2007 hydraulic stimulation at the geothermal site of Groß Schönebeck, Germany

**Figure 5:**
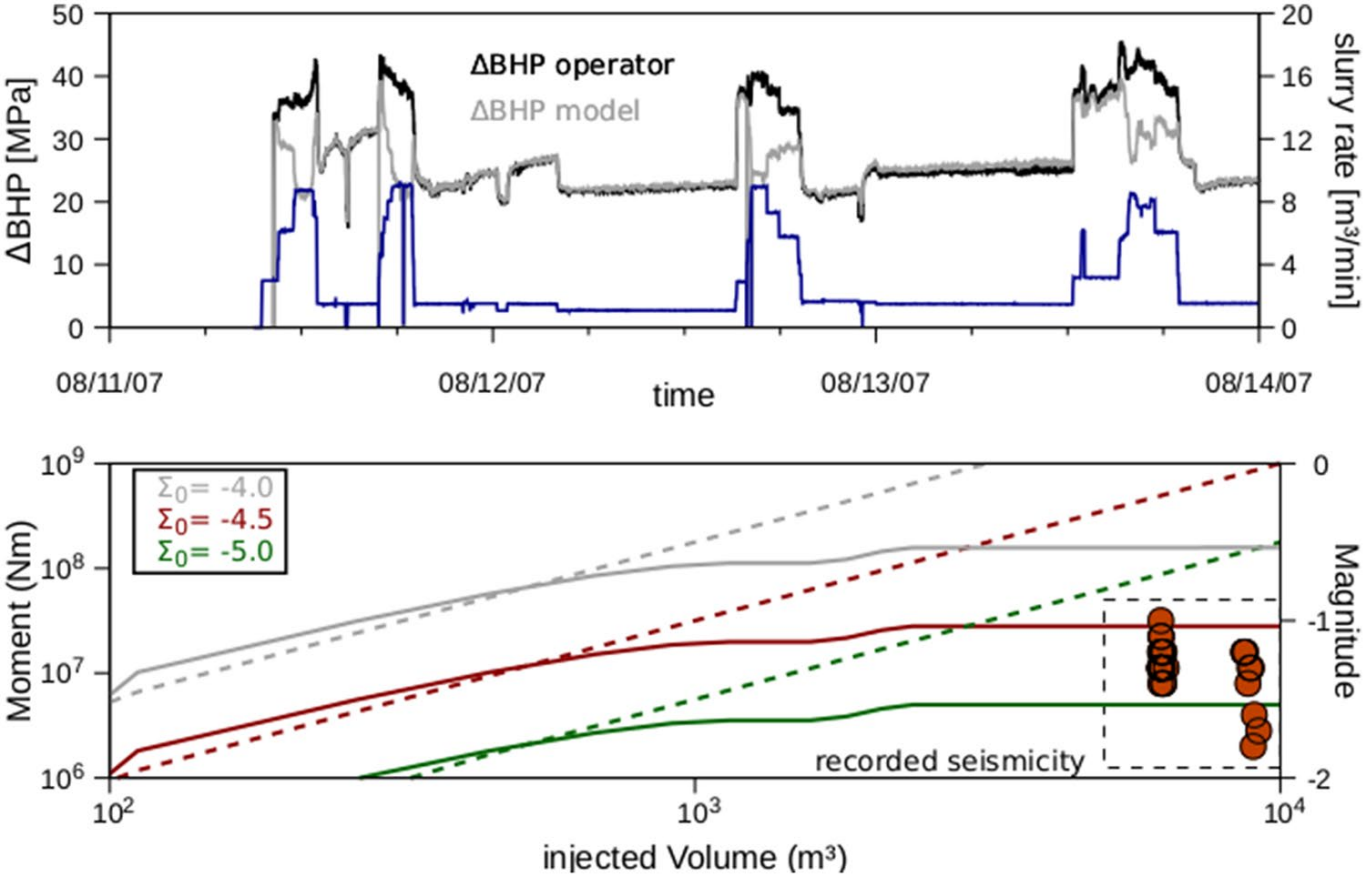
Modelling the 2007 reservoir treatment at Groß Schönebeck^22^. Upper Panel: imposed injection rates (blue curve) as well as measured (black curve) and computed (grey curve) bottom-hole overpressure ($\mathrm{\Delta BHP}$) for the 2007 stimulation experiment carried out in Groß Schönebeck. Lower panel: predicted (continuous curves) maximum magnitudes and seismic moment as a function of the total injected volume. Dashed curves are predictions derived from Eq. (14) in^10^ for different values of the tectonic seismogenic index (${\Sigma }_{0}$) that are considered reasonable for the Groß Schönebeck site^26^. The dashed rectangle encloses the recorded micro-seismicity.

In this section, we apply our model to a real-world case study and address a relatively complex stimulation experiment performed at the scientific geothermal facility of Groß Schönebeck in Northern Germany^22^. This 5-day water-frac treatment targeted the volcanic-rock section of the reservoir and consisted of five distinct cyclic-injection phases, with maximum flow rates of up to 9 m^3^/min (Fig. 5). In total, 13,170 m^3^ of slickwater and 24.4 tons of meshed-quartz sand were injected during the treatment while maintaining a wellhead pressure below 58.6 MPa (Figure S2). During the last two cycling stages, micro-seismic events with moment magnitudes ranging between − 1.8 and − 1.0 were recorded by a downhole seismometer located close to the injection section of the well. The hypocentre distribution exhibited a degree of directional ordering, with seismic activity migrating away from the injection well with time. The relocated seismic catalogue was found to be consistent with the presence of a seismic fault plane that was favourably oriented for re-activation under the in-situ stress state^39^ (Figure S3).

The aim of this modelling example is to showcase the forecast capability of our approach when applied to a complex geological set-up and injection protocol by also considering additional effects from the nonlinear physics on the resulting seismic response of the reservoir. For this purpose, we set up a 3D thermo-hydro-mechanical model of the stimulation treatment, which integrates details of the geological architecture of the reservoir, including mapped major fault zones, the open-hole section of the well, as well as the stimulated fracture. The hydraulic stimulation was considered implicitly by imposing variations in the fracture aperture as computed by a frac model^22^, which were also used to compute the evolution through time of the fracture permeability (Figure S2). Supplementary Information 3 provides a detailed description of the model set-up, including rock-properties and imposed boundary and initial conditions. The overall goal of the simulation was to match the spatio-temporal evolution of monitored micro-seismicity as well as their magnitude distribution. We identify a seismic event at a specific location following a Mohr–Coulomb-type rupture model, where rupture occurs if resolved stress magnitudes are higher than the critical yield strength of the rock. By assuming a constant friction coefficient, such critical value can be recast in term of an adimensional parameter, the slip tendency given by the ratio of shear to normal loading^39^. It follows, that an induced event will occur if the value of the slip tendency exceeds the value of the imposed static friction coefficient (considered equal to 0.6 in this study).

Model validation was done by a history matching against the bottom-hole pressure (BHP) measured during the treatment (Fig. 5, upper panel). We then used the results from the best-fitting model to compute the spatio-temporal evolution of the micro-seismic events and expected variations in computed magnitudes (Fig. 5; Figure S3). In Figure S3 we present the results of the best fitting model, in terms of the hydro-mechanical state of the reservoir after the fourth stimulation stage. We computed a maximum fluid-pressure increase of approximately $\Delta \mathrm{p}=$ 8.1 MPa along the seismic plane, higher than the level required to drive instability based on in-situ stress conditions ($\Delta \mathrm{p}=$ 6.2 MPa). We also observed no appreciable temperature changes during the entire stimulation and therefore concluded that for this stimulation, a pore pressure increase that was enhanced by poroelastic stress transfer triggered the reactivation of the fault, thereby triggering the observed micro-seismicity. Indeed, computed values of slip-tendency at the locations of the recorded hypocentres are in line with the fault instability in these locations and also match the monitored temporal pattern, with events that show an upward propagation along the seismic plane (Figure S3; Movie S1).

The model is also able to reproduce the upper bound of observed magnitudes of the induced events (Fig. 5 lower panel). Relying on a classical log-linear scaling between the total injected volume and maximum expected magnitudes^9,10^, we systematically overestimate the magnitude limits of the induced microseismicity, which occurred during the last two injection stages in Groß Schönebeck (dashed curves in Fig. 5). On the contrary, our model can capture the observed breakdown of the log-linear scaling between the injected volume and thereby to match the limits of induced event magnitudes. We interpret this result as a stress-memory effect of the reservoir caused by the operational control on the maximum wellhead pressure (Figure S2), which is similar to the Kaiser effect often discussed in global geothermal systems^28,33^.

## Discussion and conclusions

We introduced a new model that generalises the classical SI formalism^23,24^ to arbitrary physics, and provides a novel framework for forecasting induced seismicity. Our model enables reservoir physics to be integrated into a probabilistic hazard-assessment framework thereby offering improved predictability of induced and triggered earthquakes. Whether in this study we examined mainly applications for geothermal reservoirs, the flexibility of our model allows for broader applications of subsurface operations. In addition to applications in induced seismicity, our theory can also be extended for use in natural earthquakes, in which faults are loaded by a transient stress source that can be described in terms of FCS variations^40–42^.

The model provides probabilistic estimates for induced earthquake magnitudes that are in line with field observations and reconciles several injection-induced seismicity observations. In the case of pore pressure triggering only, the model reduces to the classical and widely accepted SI model for induced *seismicity^7,10,23–26,32,43^ and portrays a log-linear relation between expected maximum induced magnitudes and net injected volume (Fig. 1). We further demonstrated that seismicity rates are sensitive to the injection protocol in terms of both pressurisation rates and wellhead-pressure magnitudes. The peak in the seismicity rates scales with the highest-magnitude pressurisation rates and can therefore be effectively limited via rate stepping or cyclic tests. The injection protocol also affects the post-injection seismic behaviour, with cyclic-stimulation treatments showing faster relaxation at shut-in and therefore a lower post-injection seismic hazard than monotonic stimulations.

Considering the additional effects of thermo-poroelastic stress variations during long-term re-injection of geothermal fluid results in a larger spatial seismic footprint and increased seismic risk. The timing of these dynamics depends on the diffusive time scale for pore pressure equilibration. While seismicity is initially bounded by the pressurised front, upon pressure equilibrium, it propagates at a greater distance ahead of the pressure front due to thermo-poroelastic effects. Long-term (i.e. decades) cold-water injection—which is commonly performed in geothermal re-injection wells—is thus likely to have much greater seismic-hazard potential than is commonly assumed due to induced thermo-poroelastic stress transfer. This finding is of particular relevance for geothermal fields, which observe frequent seismicity of considerable magnitudes during regular operations^29^. Our results agree with the main conclusions derived from a recent compilation of spatial seismicity decay away from the injection well of induced seismicity worldwide^34^, which also distinguished two main populations in their dataset: pressure dominated (low poroelastic coupling) induced earthquakes characterized by near-well seismicity and abrupt decay; and, a second population exhibiting a larger spatial seismic footprint and induced magnitudes and a more gradual decay caused by poroelastic stress transfer.

The larger spatial seismic footprint associated with poroelastic and thermoelastic stress transfer in geothermal systems entails that the presence of sub- or close to critical earthquake magnitudes during the early phases (few years) of operations should be taken as an indication of an increased likelihood to trigger a potentially critical (e.g. damaging) earthquake with ongoing operations, also after shut-in. These findings have important implications on currently adopted methods—in which seismicity forecasts are derived from a projection in time of recorded seismicity and planned injected volumes—and which are thus likely to provide lower-bound estimates of the seismogenic response of the reservoir^34^ and could fail to reconcile the spatial extent of the seismic cloud, as observed, for example, in Pohang^19,20^.

In a reservoir, induced earthquakes are the direct result of nonlinear reservoir physics in combination with complex geology and large-scale tectonics. Our approach assumes a Mohr–Coulomb-type rupture model for induced earthquakes and a constant friction coefficient and does not account for the dynamics of fault healing as a function of time and slip as considered by for example a classical rate and state formalism. These effects could be considered in the underlying reservoir model, where the frictional response of a fault would affect the stress state in the reservoir domain, e.g. imposing additional variations in the computed FCS field. However, to add these dynamics would require a detailed knowledge of the geometry and frictional properties of active faults, which are hardly constrained by direct measurements in the field.

In the field, the tectonic background of seismic hazard exerts an additional influence on the resulting magnitudes that cannot be entirely mitigated by changes in injection protocols. Any risk assessment should therefore be evaluated on an individual basis and on a critical evaluation of local conditions via extensive exploration- and monitoring campaigns. In this regard, our approach allows to estimate this critical tectonic characteristic, the seismogenic index (${\Sigma }_{0}$) in our formulation, for a particular reservoir based on history matching of data derived from a stimulation treatment, which can later be used to estimate the induced seismicity by any type of stimulations, whether injection or production scenarios. Therefore, once validated against field observations and on-site monitoring, the predictions of our model can then be used to inform ATLSs as underlying model for seismicity estimation. Additionally, our model can be used to identify optimal injection strategies to mitigate potential seismic hazards from planned operations and to test the applicability of novel concepts for forecasting and controlling induced earthquake hazards.

## Supplementary Information

Supplementary Information 1.

Supplementary Video 1.

## Data Availability

All data and codes supporting the findings of this manuscript are available from the corresponding author upon reasonable request.

## References

[1] Scholtz CH (1998). Earthquakes and friction laws. Nature.

[2] Foulger GR (2018). Global review of human-induced earthquakes. Earth Sci. Rev..

[3] Shapiro SA, Dinske C, Kummerow J (2007). Probability of a given-magnitude earthquake induced by a fluid injection. Geophys. Res. Lett..

[4] Gischig VS, Wiemer SA (2013). Stochastic model for induced seismicity based on non-linear pressure diffusion and irreversible permeability enhancement. Geophys. J. Int..

[5] Kiraly-Proag E (2016). Validating induced seismicity forecast models—induced seismicity TestBench. J. Geophys. Res. Solid Earth.

[6] Schultz R, Beroza GC, Ellsworth WL (2021). A risk-based approach for managing hydraulic fracturing–induced seismicity. Science.

[7] Langenbruch C, Zoback MD (2016). How will induced seismicity in Oklahoma respond to decreased saltwater injection rates?. Sci. Adv..

[8] Zang A (2019). How to reduce fluid-injection-induced seismicity. Rock Mech. Rock Eng..

[9] McGarr A (2014). Maximum magnitude earthquakes induced by fluid injection. J. Geophys. Res. Solid Earth.

[10] van der Elst NJ (2016). Induced earthquake magnitudes are as large as (statistically) expected. J. Geophys. Res. Solid Earth.

[11] Galis M, Ampuero JP, Mai MP, Cappa F (2017). Induced seismicity provides insight into why earthquake ruptures stop. Sci. Adv..

[12] Guglielmi Y (2015). Seismicity triggered by fluid injection-induced aseismic slip. Science.

[13] De Barros L, Cappa F, Guglielmi Y (2019). Energy of injection-induced seismicity predicted from in-situ experiments. Sci. Rep..

[14] Segall P, Grasso JR, Mossop A (1994). Poroelastic stressing and induced seismicity near the lacq gas field, southwestern France. J. Geophys. Res. Solid Earth.

[15] Eyre TS (2019). The role of aseismic slip in hydraulic fracturing-induced seismicity. Sci. Adv..

[16] Majer EL (2007). Induced seismicity associated with enhanced geothermal systems. Geothermics.

[17] Wang L (2020). Laboratory study on fluid-induced fault slip behavior: The role of fluid pressurization rate. Geophys. Res. Lett..

[18] Passelègue FX, Brantut N, Mitchell TM (2018). Fault reactivation by fluid injection: Controls from stress state and injection rate. Geophys. Res. Lett..

[19] Ellsworth WL, Giardini D, Towned J, Ge S, Shinamoto T (2019). Triggering of the Pohang, Korea, Earthquake (Mw 5.5) by enhanced geothermal system stimulation. Seismol. Res. Lett..

[20] Kim KH (2018). Assessing whether the 2017 Mw 5.4 Pohang earthquake in South Korea was an induced event. Science.

[21] Kwiatek G (2015). Effects of long-term fluid injection on induced seismicity parameters and maximum magnitude in northwestern part of The Geysers geothermal field. J. Geophys. Res..

[22] Blöcher G (2015). Hydraulic history and current state of the deep geothermal reservoir Groß Schönebeck. Geothermics.

[23] Shapiro SA, Dinske C, Langenbruch C, Wenzel F (2010). Seismogenic index and magnitude probability of earthquakes induced during reservoir fluid stimulations. Lead. Edge.

[24] Shapiro SA (2018). Seismogenic index of underground fluid injections and productions. J. Geophys. Res. Solid Earth.

[25] Schultz R (2018). Hydraulic fracturing volume is associated with induced earthquake productivity in the Duvernay play. Science.

[26] Shapiro SA, Dinske C (2021). Stress drop, seismogenic index and fault cohesion of fluid-induced earthquakes. Rock Mech. Rock Eng..

[27] Parotidis M, Shapiro SA (2004). A statistical model for the seismicity rate of fluid-injection-induced earthquakes. Geophys. Res. Lett..

[28] Li C, Nordlund E (1993). Experimental verification of the Kaiser effect in rocks. Rock Mech. Rock Eng..

[29] Juncu D (2020). Injection-induced surface deformation and seismicity at the Hellisheidi geothermal field. J. Volcanol. Geotherm. Res..

[30] Maxwell SC (2013). Unintentional seismicity induced by hydraulic fracturing. CSEG Rec. Focus Artic..

[31] Buijze L (2019). Review of induced seismicity in geothermal systems worldwide and implications for geothermal systems in the Netherlands. Neth. J. Geosci..

[32] Langenbruch C, Shapiro SA (2010). Decay rate of fluid induced seismicity after termination of reservoir stimulations. Geophysics.

[33] Zang A (2021). Relaxation damage control via fatigue-hydraulic fracturing in granitic rock as inferred from laboratory-, mine-, and field-scale experiments. Sci. Rep..

[34] Goebel THW, Brodsky EE (2018). The spatial footprint of injection wells in a global compilation of induced earthquake sequences. Science.

[35] Parisio F (2019). The risks of long-term re-injection in supercritical geothermal systems. Nat. Commun..

[36] Catalli F, Meier MA, Wiemer S (2013). The role of Coulomb stress changes for injection-induced seismicity: The Basel enhanced geothermal system. Geophys. Res. Lett..

[37] Johann L, Shapiro SA, Dinske C (2018). The surge of earthquakes in Central Oklahoma has features of reservoir-induced seismicity. Sci. Rep..

[38] Jansen G, Miller SA (2017). On the role of thermal stresses during hydraulic stimulation of geothermal reservoirs. Geofluids.

[39] Blöcher G (2018). Evaluating micro-seismic events triggered by reservoir operations at the geothermal site of Groß Schönebeck (Germany). Rock Mech. Rock Eng..

[40] Stein RS, Lisowski M (1983). The 1979 homestead valley earthquake sequence, California: Control of aftershocks and postseismic deformation. J. Geophys. Res. Solid Earth.

[41] Harris R, Simpson R (1992). Changes in static stress on southern California faults after the 1992 Landers earthquake. Nature.

[42] Cruz-Atienza VM, Villafuerte C, Bhat HS (2018). Rapid tremor migration and pore-pressure waves in subduction zones. Nat. Commun..

[43] Langenbruch C, Weingarten M, Zoback MD (2018). Physics-based forecasting of man-made earthquake hazards in Oklahoma and Kansas. Nat. Commun..

