# Supplementary material for: Projecting seismicity induced by complex alterations of underground stresses with applications to geothermal systems
Source: Sci Rep. 2021 Dec 7;11:23560. doi: 10.1038/s41598-021-02857-0 (PMC8651648; doi:10.1038/s41598-021-02857-0)
Supplement: Supplementary file 1 — Supplementary Information 1. [file 41598_2021_2857_MOESM1_ESM.pdf]

Supplementary Information to the manuscript "Projecting seismicity induced by complex alterations of underground stresses with applications to geothermal systems" by M. Cacace, H. Hofmann, and S.A. Shapiro

This supplementary-materials file contains a detailed derivation of the modified GR statistics for induced seismicity (Supplementary Information 1); a derivation of the conservation equations for thermo-hydro-mechanical reservoir numerical analysis together with a description of the synthetic-reservoir-model setup discussed in the main text (Supplementary Information 2); a detailed description of the model setup for the 2007 stimulation performed in Groß Schönebeck (Supplementary Information 3); and, a derivation of the modified Omori's law for post-injection seismicity (Supplementary Information 4).

We also include additional figures where we compute the cumulative hydraulic energy input for the different injection protocols discussed in the main text (Figure S1), and additional informative materials on the Groß Schönebeck simulation (Figures S2 and S3).

Supplementary Table 1 lists all material properties considered in the study, and we also include a movie that illustrate the spatio-temporal evolution of the computed micro-seicmic events for the Groß Schönebeck simulation (Movie S1).

Supplementary Information 1. Derivation of modified GR statistics for induced earthquakes

In order to render classical GR statistics applicable to induced seismicity, we considered frictional Coulomb stress changes $(\delta \mathrm{FCS})$ when mapping instability within a Mohr–Coulomb (MC) failure criterion as:

(S1.1) $$\delta \mathrm{FCS} = \delta\tau - \mu(\delta\sigma - \delta p)$$

In equation (S1.1), $\delta\tau$, $\delta\sigma$, and δp are variations in shear stress, normal stress, and pore pressure, respectively, and μ is the friction coefficient.

Equation (S1.1) provides a description of instabilities that can be integrated into any reservoir-modelling study. The main benefit of this model is its ability to consider the role of (thermo-poro)elastic stress transfer – that is, the dynamics responsible for communicating a developed stress perturbation (origin) to a particular fault (receiver). Upon a closer inspection of equation (S1.1), three distinctive – albeit mutually interacting – stress components can be distinguished in the model: variations in (i) shear stress, (ii) (effective) normal stress, and (iii) the friction coefficient. If we assume a constant tectonic stressing rate and friction coefficient, the main stress parameter that affects

the evolution of Coulomb stress is provided by pore pressure variations – that is, variations in Coulomb stress scale with pore pressure variations. Therefore, the predictions from the $\delta\mathrm{FCS}$ model closely match those obtained from seismicity-rate approaches based on a (non-)linear diffusion front concept**[1,2,3]**.

We follow previous work in integrating equation (S1.1) into a statistical framework**[3,4]** and therefore consider injection inside a porous domain that consists of a set of randomly oriented, non-interacting point-like defects (fractures). The volume concentration of the defects (ζ) is statistically homogeneously distributed. Each fracture is characterised by a critical-failure state, as exemplified by a critical value of $\delta\mathrm{FCS}$ for a slip event to occur, which is consistent with MC failure theory. The formulation is therefore consistent with Biot's theory of poroelasticity and disregards the stricter assumption that pore pressure increase/relaxation causes induced seismicity. The critical value of $\delta\mathrm{FCS}$ is characterized by its probability density function, $w(C)$. Then, the probability that an event will occur within the time interval from the start of the stimulation untill the given moment (t) reads as:

(S1.2) $$W_{ev}(\delta FCS, t) = \int_0^{M[\delta FCS(\mathbf{x},t)]/sin(\phi)} w(C)\ dC$$

Equation (S1.2) uses the concept of the minimum positive monotonic majorant,

defined as the least majorant limited to the positive half-axis in the space of the function values for a non positive defined function, of Coulomb stress variations $M[\delta FCS(\boldsymbol{x},t)]$ as a variable of interest.

The above-mentioned probability-density function, w(C), can be approximated by a box-car function with local support, with an upper (Cmax) and lower (Cmin) bound. The lower bound of the distribution is taken to coincide with tidal loads ($\mathrm{Cmin} \sim 10^3$ MPa), while the upper bound is usually considered to be greater than tens of MPa**[5]**. We can therefore approximate the probability function as $w(C) \sim \frac{1}{Cmax}$. The cumulative number of induced earthquakes at a given time is given by the product of the probability that an event will occur times the volume concentration of defects in the stimulated volume:

(S1.3) $$N_{ev}(t) = \zeta \int_{\mathrm{V}} W_{ev}(\delta FCS, t) dV = \frac{\zeta}{\mathrm{Cmax}} \int_{\mathrm{V}} \frac{M[\delta FCS(\boldsymbol{x},t)]}{\sin(\phi)} dV$$

Equation (S1.3) can be easily extended to describe the cumulative number of induced earthquakes with a magnitude greater than a given one as

(S1.4) $$N_{\geq M}(t) = W_{\geq M}\, N_{ev}(t) = W_{\geq M}\, \frac{\zeta}{\sin(\phi)\ \mathrm{Cmax}} \int_{\mathrm{V}} M[\delta FCS(\boldsymbol{x},t)] dV\ ,$$

where $W_{\geq M}$ is the cumulative probability that a particular event will exceed a

given magnitude M. The latter probability is drawn according to a GR frequency–magnitude statistics**[3]**:

(S1.5) $$log_{10}(W_{\geq M}) = a_w - bM$$

Combining equations (S1.4) and (S1.5) yields the final expression of the modified GR statistics for induced seismicity. In order to be consistent with the formulation adopted in the main text, the final equation is formed using the tectonic seismogenic index ($\Sigma_0 = log_{10}(\frac{\zeta}{S\ Cmax}) + a_w$)**[3,4]**:

(S1.6) $$log_{10}(N_{\geq M}(t)) = [\Sigma_0 + \delta\Sigma(t)] - bM$$

The final expression for $\delta\Sigma(t)$ is provided in equation (4) in the main text.

Supplementary Information 2. Thermo-hydro-mechanical reservoir modelling – Governing equations and model setup

Here, we describe the balance equations for fluid mass, energy, and momentum for a porous medium that are relevant in thermo-hydro-mechanical (THM) reservoir applications. More details on the derivation of the system of equations, their numerical implementation, and their extensions (which account for diverse inelastic deformation mechanisms) can be found in**[6,7,8]**.

The following primary variables are used to derive the basic equations: temperature (T), pore pressure (p), and the solid displacement vector ($\mathbf{u} = \{u_x, u_y, u_z\}$).

The mass balance of the porous rock is expressed as a volumetric average of its fluid- and solid components:

$$\text{(S2.1)}\quad \frac{\varphi}{\rho_f} D_t^f \rho_f + \frac{(1-\varphi)}{\rho_s} D_t^s \rho_s + \nabla \bullet \mathbf{v}_s + \nabla \bullet \mathbf{q} = 0$$

In equation (S2.1), φ is the porosity; ρ_f and ρ_s are the fluid- and solid density, respectively; $\mathbf{v}_s$ is the solid deformation velocity; $\mathbf{q} = \varphi(\mathbf{v}_f - \mathbf{v}_s) = -\frac{k}{\mu_f}(\nabla p - \rho_f \mathbf{g})$ is the Darcy velocity; and the operators $D_t^i\,() = \frac{\partial}{\partial t}() + \mathbf{v}_i \bullet \nabla\,()$ are the total (Lagrangian) derivatives with respect to a moving fluid (i = f) and solid (i = s). It is possible to re-write equation (S2.1) in terms of the primary variables using the following considerations**[6]**:

The first term on the left side of equation (S2.1) can be re-written as

$$\text{(S2.2)}\quad D_t^f \rho_f = \varphi\ (\frac{1}{K_f} D_t^f p - \beta_f D_t^f T),$$

where T denotes temperature, K_f is the reciprocal of the fluid compressibility, and β_f is the thermal-expansion coefficient of the fluid. Re-writing the second

term in equation (S2.1) requires defining a proper constitutive mechanical model, which can be derived from linear-momentum conservation ($\nabla \bullet (\sigma - \alpha p \mathbf{I}) + \rho_b \mathbf{g} = 0$) and – under the assumption of linear Biot poroelasticity – it reads as

(S2.3) $\dot{\sigma}_{ij} = C_{ijkl} \dot{\epsilon}^{e}_{kl}$,

with C_{ijkl} as the elastic-stiffness tensor.

By considering equations (S2.2) and (S2.3) and disregarding second-order non-linear advective terms, equation (S2.1) can be rewritten as

(S2.4) $\frac{1}{M_b} \frac{\partial p}{\partial t} - \beta_b \frac{\partial T}{\partial t} - (1 - \alpha) \dot{\epsilon}^{e}_{kk} + \dot{\epsilon}_{kk} + \nabla \bullet \mathbf{q} = 0$

In equation (S2.4), M_b is the Biot modulus, β_b is the bulk thermal-expansion coefficient, and α is the Biot poroelastic constant. The following relation between solid velocity and total volumetric strains is used: $\nabla \bullet \mathbf{v}_s = \nabla \bullet \dot{\mathbf{u}} = \dot{\epsilon}_{kk}$.

Equation (S2.4) expresses the mass balance for a porous medium and neglects any irreversible deformation mechanism – that is, $\boldsymbol{\epsilon} = \boldsymbol{\epsilon}^{e}$. If a small-strain formulation is assumed, it is then possible to split the total strain tensor ($\boldsymbol{\epsilon}$) into the sum of an elastic reversible strain component ($\boldsymbol{\epsilon}^{e}$) and an inelastic

irreversible strain component ($\boldsymbol{\epsilon}^{in}$), which leads to the generalisation of equation (S2.4):

$$\text{(S2.5)} \quad \frac{1}{M_b}\frac{\partial p}{\partial t} - \beta_b \frac{\partial T}{\partial t} + \alpha \dot{\epsilon}_{kk} + (1-\alpha)\dot{\epsilon}_{kk}^{in} + \nabla \bullet \mathbf{q} = 0$$

Following a similar approach, an evolution equation can be derived for the system porosity:

$$\text{(S2.6)} \quad \frac{\partial \varphi}{\partial t} = \frac{(\alpha - \varphi)}{K_s}\frac{\partial p}{\partial t} - \beta_b \frac{\partial T}{\partial t} - (\alpha - \varphi)\dot{\epsilon}_{kk} + (1-\alpha)\dot{\epsilon}_{kk}^{in}$$

The internal energy balance of the porous medium under thermal equilibrium between the solid- and fluid phase and that considers additional thermo-elastic stress is thus

$$\text{(S2.7)} \quad \frac{\partial \rho_b c_b T}{\partial t} + T_0 \beta_b \dot{\epsilon}_{kk}^{e} + \nabla \bullet (\rho_f c_f \mathbf{q} T - \lambda_b \nabla T) - \dot{H} = 0,$$

where T_0 is the absolute temperature of the porous medium in a stress-free state and the subscript b stends for bulk properties. For example, $c_b = \varphi c_f + (1-\varphi)c_s$ is the heat capacity, $\lambda_b = \varphi \lambda_f + (1-\varphi)\lambda_s$is the thermal

conductivity, $\beta_b = \varphi\beta_f + (1-\varphi)\beta_s$ is the thermal-expansion coefficient, and $\dot{H}$ is the internal heat-production rate. For simplicity, we disregard thermal effects resulting from fluid dilation or shear heating in equation (S2.7)**[6,7]**.

In the present study, we numerically solve the system of equations outlined above via the finite element method (FEM), as implemented in the open-source software Golem**[6]**. In what follows, we describe the model setup for the synthetic experiments as discussed in the main text, and we dedicate Supplementary Information 3 to an in-depth description of the model setup for the 2007 Groß Schönebeck stimulation.

For these synthetic cases, we built a representative 3D model of a typical geothermal system. The model geometry is defined by a 5 km x 5 km x 1 km reservoir, for which we target a depth of approximately 4 km below sea level. The reservoir is subjected to a tectonic stress field resembling a normal-faulting regime – that is, $\sigma_{zz} = \sigma_1 > \sigma yy = \sigma_2 > \sigma_{xx} = \sigma_3$, as expected at the targeted depth. The imposed lithostatic loading corresponds to an average overburden solid density of 2,500 kg/m^3, and we consider a stress ratio between the minimum and maximum horizontal stress of 0.65. We also consider a no-flow boundary condition along the base of the model and hydrostatic pressure conditions at the sides and along the top surface (open boundaries). The thermal boundary conditions are a constant basal heat flow (60 mW/m^2) and a fixed temperature following a geothermal conductive gradient of 33 °C/km

along the top surface. Fluid- and rock properties are listed in Supplementary Table 1. Please note that we tested a range of values for the main properties within bounds that are representative of low- to middle-enthalpy hydrothermal reservoirs, and we found that the main conclusions of our study are robust to the tested variations. Injection/production is modelled by a point source, for which we prescribe a given inflow/outflow rate that is either constant or variable in time according to the specific case considered. We locate the injection/production source points in the middle of the model in order to avoid boundary effects during the simulation. We derive proper initial conditions for the pore-fluid-pressure-, temperature-, and stress distribution by solving for a steady-state simulation in a prior initialisation phase. For all cases considered, we initialise the transient stage – which consists of either injection or circulation – using the pressure-, temperature-, and stress distribution obtained by the steady-state numerical experiment.

Supplementary Information 3. Water-frac treatment at Groß Schönebeck

The geothermal facility of Groß Schönebeck (in Northern Germany) is considered a prototypical low- to medium-enthalpy geothermal reservoir. The targeted reservoir horizons comprise interbedded layers of siliciclastic rock (upper Rotliegend formation) that lie atop a volcanic lower-Permian sequence at a depth of around 3,800–4,350 m below sea level. As a result of a seismic campaign and available wellbore data, a detailed three-dimensional geological

model of the reservoir has been made available**[9]**. The model covers an area of approximately 4 km x 4 km in lateral extension and features all reservoir horizons and major faults mapped in the area. The stress regime – which was estimated via field measurements – was found to vary with depth in the reservoir between a preferential normal regime and a strike-slip fault regime. The geothermal facility consists of a doublet system in which both wells underwent several hydraulic and gel-proppant stimulation treatments. Of interest for this study is the latest hydraulic stimulation, which was performed in the volcanic section of the production well in 2007 (frac interval: 4,350–4,404 m) with the goal of increasing the area of fluid uptake via a stimulated fracture. The stimulation was carried out over a period of approximately five days and consisted of five daily cycles with flow rates of up to 9 m^3/min. In order to limit wellhead-pressure build-up in excess of a safety threshold (considered to be 58.6 MPa), a friction-reducing agent was used during the high-flow-rate cycles (Figure S2). During the entire stimulation treatment, a total of 13,170 m^3 of slickwater and 24.4 tons of meshed-quartz sand were injected in order to support the fracture opening (Figure 5, upper panel in the main text). Micro-seismicity mainly occurred after the final two pressure cycles (the fourth and fifth cycle), with moment magnitudes ranging from between -1.8 and -1.0**[9]**.

Using all available data, we set up a 3D thermo-hydro-mechanical model of the stimulation treatment in a similar fashion, as described in**[10]**. The model

integrates details of the geological architecture of the reservoir, including mapped major fault zones, the open-hole section of the well, as well as the stimulated fracture.

We imposed open hydraulic and thermal boundary conditions along the sides and the top surface, whereas a no-flow boundary was applied at the base of the model, and we imposed a basal heat flow of 65 mW/m^2. Lithostatic loading conditions were imposed at the top of the model, and we applied roller boundary conditions for the displacement along the base of the model. We used the in-situ stress state as measured in the reservoir to calibrate the values of the imposed kinematic boundary conditions along the model sides, and we therefore performed a first numerical analysis under steady-state conditions in which we systematically varied the values of the imposed normal displacement along the side boundaries of the model in order to match the in-situ stress field (which transitions from strike-slip to normal faulting). In the following stage, we carried out a transient analysis of the 5-day stimulation by initialising the model with the pressure-, temperature-, and displacement values obtained from the previous steady-state simulation. In the transient simulation, we imposed the applied flow rate via linear integration over the open-hole section of the stimulation well (Figure 5 in the main text). In order to consider variations in the mechanical and fluid properties of the stimulated fracture, we computed the evolution of its hydraulic and mechanical aperture based on the applied flow rate and resulting wellhead pressure (Figure S2), and we used these values to

compute variations in fracture permeability during the stimulation via a cubic-law correlation that assumed laminar flow conditions in the fracture. We performed history matching of the bottom-hole pressure (BHP) measured during the treatment that considered the bulk permeability of the volcanic section of the reservoir as the variable of interest (Figure 5). The best-fitting model was then used to compute variations in seismicity rates and magnitudes, as presented in the main text (Figure 5).

Supplementary Information 4. Post-injection seismicity and Omori's law

One limitation of classical approaches to induced seismicity is their inability to provide a theoretical description of post-injection seismicity. Increasing evidence from several hydraulic stimulation treatments from around the world suggests that the largest-magnitude events can also occur shortly after the termination of operations and can only be somewhat controlled by current seismic-risk-mitigation strategies (i.e. traffic light systems). Based on an extensive catalogue of induced earthquakes and numerical and theoretical computations of pore-pressure-relaxation dynamics, previous work**[11]** has advanced the hypothesis that the decay of post-injection seismicity can be approximately described analogously to the decay of aftershock activity following a main event**[12,13]**. **[11]** presented a modified Omori's law in order to provide a simple empirical formula for computing the number of induced events of a given magnitude that are applicable to the post-injection stage:

$$(S4.1)\quad N_{(\geq M)}(t > t_0) = \frac{1-(\frac{t}{t_0})^{(1-p)}}{p-1} N_{(\geq M)}(t = t_0)$$

In equation (S4.1), $N_{(\geq M)}(t > t_0)$ describes the number of cumulative events after shut-in, and $N_{(\geq M)}(t = t_0)$ describes the number of cumulative events at shut-in. The power-law exponent (p) for induced seismicity varies over a relatively wide range from between p = 2 and p = 10**[11]**. The modified Omori's law has been successful in explaining temporal variations observed in Oklahoma waste-water-injection wells and at the Fenton Hill (New Mexico) EGS site**[11]**. In our formulation, the dynamic triggering of seismic events in space and time is dictated by the ability of the reservoir to relax induced variations in FCS, and these variations also control post-injection seismicity rates.

SI References:

1) McGarr, A. Maximum magnitude earthquakes induced by fluid injection. *J. Geophys. Res. Solid Earth*, **119**, 1008–1019 (2014).

2) van der Elst, N. J. et al. Induced earthquake magnitudes are as large as (statistically) expected. *J. Geophys. Res. Solid Earth*, **121**, 4575–4590 (2016).

3) Shapiro, S. A., Dinske, C., Langenbruch, C., Wenzel, F. Seismogenic index and magnitude probability of earthquakes induced during reservoir fluid

stimulations. *The Leading Edge*, **29(3)**, 304-309 (2010).

4) Shapiro, S. A. Seismogenic index of underground fluid injections and productions. *J. Geophys. Res. Solid Earth*, **123(9)**, 7983-7997 (2018).

5) Shapiro, S. A., Krüger, O.S, Dinske, C. Probability of inducing given-magnitude earthquakes by perturbing finite volumes of rocks, *J. Geophys. Res. Solid Earth*, **118**, 3557-3575 (2013).

6) Cacace, M. & Jacquey, A. B. Flexible parallel implicit modelling of coupled thermal–hydraulic–mechanical processes in fractured rocks. *Solid Earth*, **8**, 921–941, (2017).

7) Jacquey, A. B. & Cacace, M. Multiphysics Modeling of a brittle-ductile lithosphere: 1. Explicit visco-elasto-plastic formulation and its numerical implementation. *J. Geophys. Res. Solid Earth*, **125**, e2019JB018474 (2020).

8) Jacquey, A. B. & Cacace, M. Multiphysics modeling of a brittle-ductile lithosphere: 2. Semi-brittle, semi- ductile deformation and damage rheology. *J. Geophys. Res. Solid Earth*, **125**, e2019JB018475 (2020).

9) Blöcher, G., et al., Hydraulic history and current state of the deep geothermal reservoir Groß Schönebeck. *Geothermics*, **63**, 27-43 (2015).

10) Blöcher, G. et al. Evaluating Micro-Seismic Events Triggered by Reservoir Operations at the Geothermal Site of Groß Schönebeck (Germany). *Rock Mech Rock Eng*, **51**, 3265–3279 (2018).

11) Langenbruch C. & Shapiro, S.A. Decay rate of fluid induced seismicity after termination of reservoir stimulations. *Geophysics*, **75(6)**, 53–62 (2010).

12) Omori, F. On the aftershocks of earthquakes. *Journal of the College of Science, Imperial University of Tokyo*, **7** ,111–200 (1894).

13) Utsu, T. A statistical study of the occurrence of aftershocks. *Geophysical Magazine*, **30**, 521–605 (1961).

Supplementary Figures

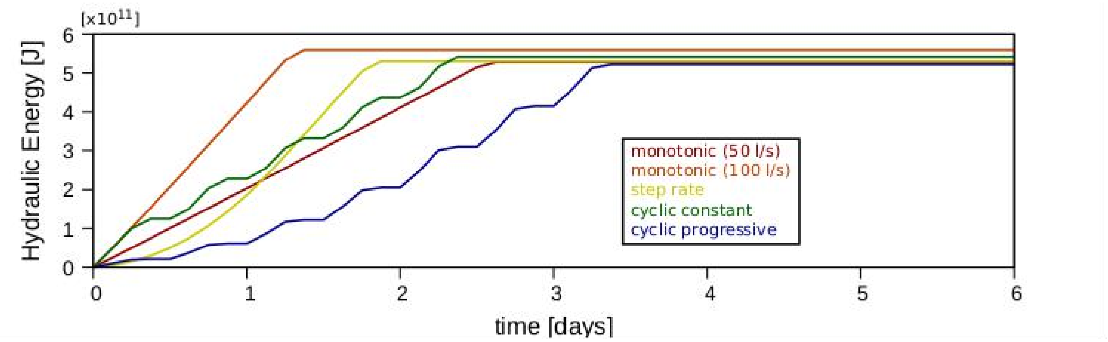

Figure S1: Input cumulative hydraulic energy for the different tested injection protocols (see Figure 2 in the main text).

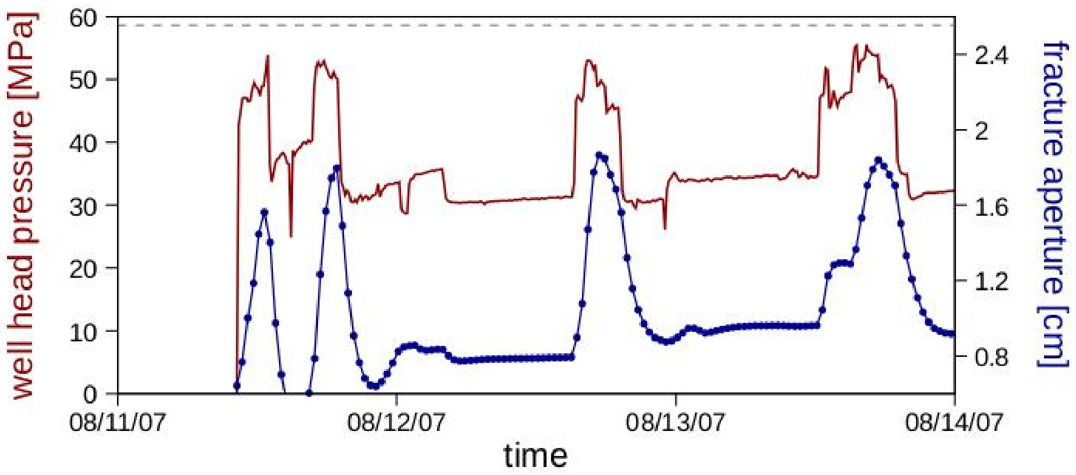

Figure S2: Monitored wellhead pressure (red curve) for the water-frac treatment performed in Groß Schönebeck[9,10]. The blue curve indicates variations in the computed mean aperture of the stimulated fracture that were used to calculate the permeability of the stimulated fracture. The dashed grey curve indicates the value of the wellhead pressure, which was considered an upper safety bound during the stimulation (~58.6 MPa).

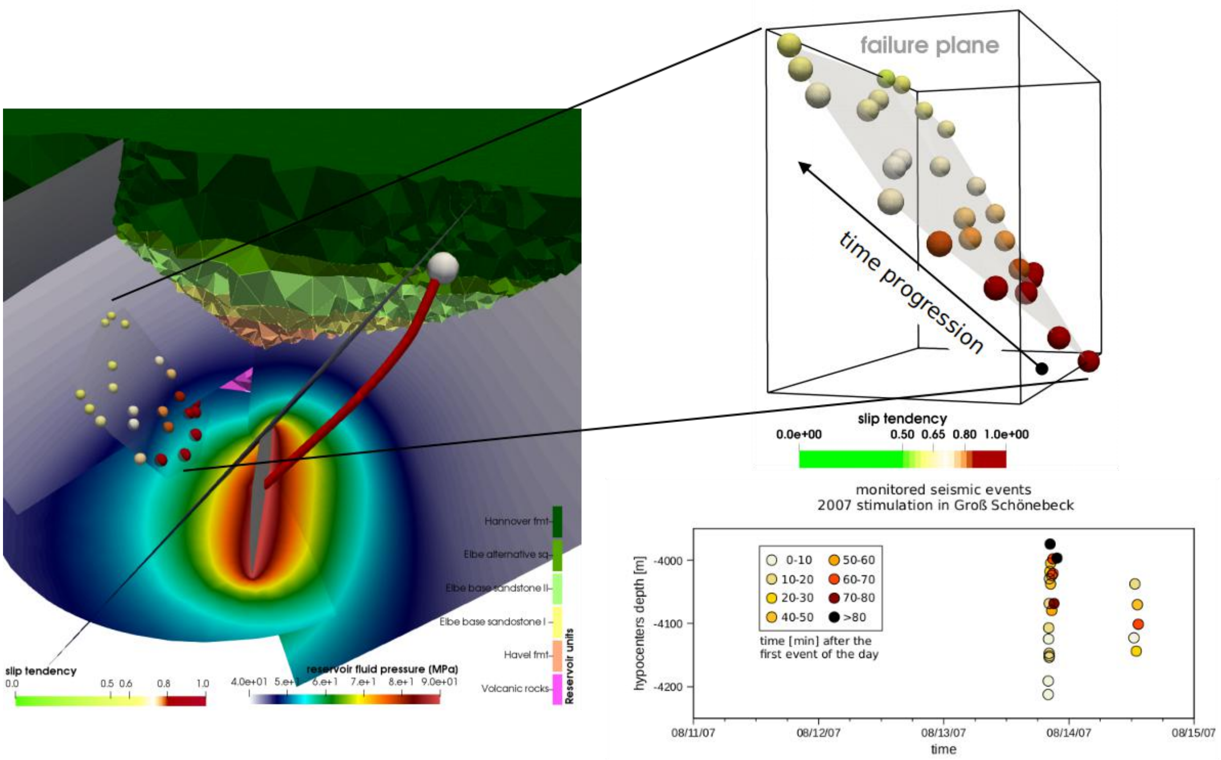

Figure S3: Left: hydro-mechanical state of the reservoir after the fourth cycle of the 2007 stimulation in Groß Schönebeck. We plot variations in pore pressure (background colours) together with the computed value of the slip-tendency ratio (i.e. the ratio between shear- and effective normal stress) for each monitored seismic event (coloured circles). The failure plane is indicated by grey shading. Right: enlargement of the seismic plane showing the location of

the monitored micro-seismic events, which are colour coded based on computed values of the slip-tendency ratio, together with a plot of the monitored spatio-temporal evolution of the microseismicity.

Supplementary Table 1: Fluid- and rock properties used for the synthetic cases presented in the main text. The range of variations in the tested properties (upper row) and their mean values (lower row) are also presented.

Properties											
Solid								Fluid			
φ [-]	k [m²]	c_s [$Wkg^{-1}K^{-1}$]	λ_s [$Wm^{-1}K^{-1}$]	β_s [K^{-1}]	K_s [GPa]	K [GPa]	ν [-]	c_f [$Wkg^{-1}K^{-1}$]	λ_f [$Wm^{-1}K^{-1}$]	β_f [K^{-1}]	K_f [GPa]
[0.1-0.3]<br>0.2	[10^{-15}- 10^{-13}]<br>10^{-14}	[900 - 1000]<br>950	[2-3]<br>2.5	[10^{-6} - 10^{-4}]<br>10^{-5}	70	60	0.25	1000	0.65	10^{-6}	2.5

Supplementary Movie: Movie S1 is an animation of the modelled seismicity (time and spatial evolution of computed single events) as computed for the 2007 hydraulic stimulation performed in Groß Schönebeck.
